# How well do adolescents recall use of mobile telephones? Results of a validation study

**DOI:** 10.1186/1471-2288-9-36

**Published:** 2009-06-12

**Authors:** Imo Inyang, Geza Benke, Joseph Morrissey, Ray McKenzie, Michael Abramson

**Affiliations:** 1 Department of Epidemiology & Preventive Medicine, School of Public Health & Preventive Medicine, Monash University, Melbourne, Australia; 2 Australian Centre for Radiofrequency Bioeffects Research, Melbourne, Australia; 3 Corporate EME Research Laboratory, Motorola Florida Research Laboratories, Fort Lauderdale, Florida, USA; 4 Department of Pharmaceutical Sciences, Nova South-eastern University, Fort Lauderdale-Davie, Florida, USA

## Abstract

**Background:**

In the last decade mobile telephone use has become more widespread among children. Concerns expressed about possible health risks have led to epidemiological studies investigating adverse health outcomes associated with mobile telephone use. Most epidemiological studies have relied on self reported questionnaire responses to determine individual exposure. We sought to validate the accuracy of self reported adolescent mobile telephone use.

**Methods:**

Participants were recruited from year 7 secondary school students in Melbourne, Australia. Adolescent recall of mobile telephone use was assessed using a self administered questionnaire which asked about number and average duration of calls per week. Validation of self reports was undertaken using Software Modified Phones (SMPs) which logged exposure details such as number and duration of calls.

**Results:**

A total of 59 adolescents participated (39% boys, 61% girls). Overall a modest but significant rank correlation was found between self and validated number of voice calls (ρ = 0.3, P = 0.04) with a sensitivity of 57% and specificity of 66%. Agreement between SMP measured and self reported duration of calls was poorer (ρ = 0.1, P = 0.37). Participants whose parents belonged to the 4^th ^socioeconomic stratum recalled mobile phone use better than others (ρ = 0.6, P = 0.01).

**Conclusion:**

Adolescent recall of mobile telephone use was only modestly accurate. Caution is warranted in interpreting results of epidemiological studies investigating health effects of mobile phone use in this age group.

## Background

The health effects of mobile (cellular) telephony are currently attracting a great deal of attention both in the mass media as well as the epidemiological literature. This increased scrutiny may be attributed to the increasing uptake of mobile telephones by the population including children and adolescents, whose developing nervous systems may be more sensitive to radiofrequency (RF) radiation emitted by mobile phone handsets and associated radio-base stations (RBS)[1]. During conventional mobile telephone voice use, most of the RF radiation is concentrated in the head leading to concerns of possible adverse health effects in the head such as development of brain tumours [2-17], altered brain electrical activity [18,19] and cognitive effects especially in children[20,21]. In 2005 the World Health Organisation EMF research agenda[22] was revised based on the conclusion that research to date failed to reveal any adverse findings of mobile phone use. Importantly, the revision also identified new areas of research interest including categorising research in children and adolescents to be a high priority.

Most studies investigating the health effects of mobile telephone use have utilized the case-control design to investigate associations of phone use and brain tumours in adult populations. However the CEFALO[23] study is investigating possible associations of brain tumours and mobile telephone use in children and adolescents. These studies generally used questionnaires which relied on participants' retrospective reconstruction of mobile phone use. However recall of exposure parameters such as number and duration of calls is generally acknowledged to be imprecise[24,25] and affected by recall bias resulting in exposure misclassification [26].

Exposure misclassification has been described as the Achilles heel of epidemiological studies that rely on participants' self-reports[27]. There are also suggestions that the precision of self-reports may be influenced by respondents' desire to conform to societal norms, making such reports unreliable and misleading[28]. The imprecision of participants' recall of exposures concerns epidemiologists and may have contributed to recent calls that all mobile telephone health effects studies relying on participants' recall should be further validated[29]. Although necessary, such validation has seldom been achieved in epidemiological studies to date.

A few studies have sought to validate recall of mobile telephone use by adults. The participants in these studies were mostly drawn from colleagues, friends and acquaintances of investigators and may not be representative of the original cohort[25,30-32] apart from one study which offered free airtime incentives[33]. Another independent recent report investigated agreements between self reports and Software Modified Phone (SMP) validated results in mobile telephone company employees[34]. The SMP is an ordinary mobile telephone incorporating additional software to record important dosimetric parameters including the number and duration of calls. The direct implication of these methodological limitations is to challenge the interpretation of these validation studies given the lack of relationship between main study and validation participants.

Whilst there are a number of cross-sectional studies that investigated health effects of mobile telephone use in children and adolescents, long term studies in this population are few. A recent Belgian longitudinal study reported poorer health including sleep deprivation in adolescent mobile telephone users[35]. However there is no published study validating mobile telephone use in children and adolescents.

Here we report the results of a cross-sectional analysis of a sample – the first validation study in adolescents utilizing software modified phones (SMP) within the same epidemiological cohort investigating the cognitive effects of mobile telephone use in adolescents.

## Methods

### Setting and population

This validation study was carried out within the broader Mobile Radiofrequency Phone Exposed Users Study (MoRPhEUS) – a prospective cohort study investigating cognitive effects of mobile telephone use in Australian secondary school students. For the main MoRPhEUS study, we invited 479 Year 7 students and eventually recruited 317 participants from 20 Melbourne secondary schools representing the three school sectors in Australia: Government, Catholic and Independent[36]. Melbourne is a cosmopolitan city, the official language is English, but almost a third (32%) of the population speaks languages other than English at home according to the Australian Bureau of Statistics 2006 census figures.

### Recruitment of Participants

Permission for students to participate in this validation study utilizing SMPs was obtained from the schools, as well as parents/guardians of students who owned and were the only users of their mobile telephones. To determine registered ownership of mobile telephones, a brief face-to-face interview was conducted. Potential participants were asked: "When your telephone bill arrives, does it bear your name?" Parents also answered a simple questionnaire about their children. Relevant questions included date of birth of child, gender, languages other than English spoken at home, how much of a health risk parents viewed mobile phones and importantly whether they consented to their child using the SMP. Students whose parents/guardians consented were also required to give their own informed consent. A recent study found most children expected some level of parental input, but thought the final decision to participate in a study should be theirs[37]. Approval was also obtained from the Standing Committee on Ethics in Research Involving Humans (SCERH) at Monash University.

Subsequently invitation to participate in this validation study was extended to students who took part in the main study and also satisfied the strict inclusion criteria determined a priori and summarized in appendix 1.

Parental Socioeconomic Status (SES) was assessed by residential postcode linked to Socio-Economic Indexes for areas (SEIFA) which is maintained by the Australian Bureau of Statistics. SEIFA 2001[38] was current at study period and provided 4 summary indexes ranging from the most disadvantaged to the most advantaged based on geographic location. We assessed possible influence of parental SES on adolescent recall of mobile telephone use with the index of Advantage/Disadvantage divided into 5 quintiles ranging from most disadvantaged (1^st ^quintile) to most advantaged (5^th ^quintile). No gratuities were offered at any stage of the recruitment process to induce participation.

### Validation instrument quality control

Although the SMPs have been previously utilized in validation studies in adult populations[25,30], the quality control measures necessary for a successful study were not reported. However the SMP is an important research resource currently being shared amongst international collaborative as well as single centre studies relevant to RF health effects research, some of which involved children and adolescents. Most of these countries operate at different radio-communication frequencies as well as RF transmissions. Thus we instituted and followed the quality control checks summarized in table 1 as part of the study protocol.

**Table 1:** SMP quality control checks

Action	Comments
Ensured that a few extra phones over the number indicated on initial visit were available and fully charged	Need for extra phones desirable as some children with valid consent may have been absent on initial visit date

Ensured adequate numbers of chargers and adaptors were available and functional	SMPs are shared around the globe from one electrical system to another. In our case the adaptors were necessary to convert to Australian electrical system

Reset the operational frequency of the phones to 900/1800 MHz or as appropriate	900/1800 MHz frequencies were the prevalent frequencies in Australia at study time. This step is very important as the present generation of SMPs is shared amongst various international research centres and operational frequencies do vary from region to region. Most of the SMPs were delivered to us with frequencies set at 1900 MHz. If not changed could suggest a phone fault and can negatively effect participation.

Ensured data collection rate was the same for all phones	Heterogeneous data collection rate can potentially affect exposure allocation of participants and hence introduce bias in a study. In this study data collection rate was uniformly set at 2.5 seconds.

Take the responsibility of Swapping SIM card from participant phone to SMP at beginning and end of use	Participants should not be saddled with this responsibility as problems encountered at this stage could potentially jeopardize participation

Provide participants with a dedicated telephone help number and e-mail address	This service is vital and ensures that participants get prompt help especially with phone and charger faults. Participants may also need to contact investigators at short notice if changing addresses suddenly such as unplanned travel including permanent relocation.

### Self reported phone use

Self reported exposure to mobile phones was assessed using a questionnaire adapted from INTERPHONE to suit the ages and local sensitivities of our participants. The relevant questions required students to volunteer information on use and ownership of mobile telephones as well as number and duration of calls made (outgoing) and received (incoming) per week expressed as digits or a range.

### Validation of Phone use

Validation of participants self reports was performed using GSM type SMPs (Motorola Timeport model: 92621XWXEA) that logged data such as number and duration of calls using a 2.5 second data collection rate. SMP data required specialised software to download and was therefore safe from manipulation by the participants. Actual phone use was logged for one week and compared with self reports over the same period.

### Statistical analysis

The distributions of self reported and logged number of mobile phone voice calls per week were skewed and normalised by logarithmic transformations prior to most analyses. Self reports and logged results were treated both as continuous and categorical variables. Agreement between categories of number of voice calls was assessed as sensitivity, specificity, positive predictive value, negative predictive value with SMP records as the gold standard. The median number of voice calls per week in the MoRPhEUS study was used as the cut-off in determining high/low exposure allocation used in these analyses. Sensitivity in this context measured the ability of participants to accurately recall their level of mobile telephone use as high when they were actually high users and specificity measured the ability of participants to report use as low when they were clearly low users. A graphical method [39] was used to assess the level of agreement and associated 95% limits between self report and SMP records. Limits of agreement were calculated as Mean difference ± 1.96 standard deviations of differences. Possible confounding influences on recall were assessed using Spearman's rank correlation coefficient (ρ). The statistical package used for all analyses was Stata version 9.0[40].

## Results

### Participation

Twelve schools randomly selected from the 20 that constituted the main MoRPhEUS cohort participated in this validation study. From these 12 schools, invitations were extended to 159 students who participated in the main study and owned mobile phones. Almost half (47%) volunteered to participate. However 15 of these students were subsequently excluded due to technological incompatibility, in that 10 owned third generation (3G) phones while SMPs used in this study only supported earlier versions and the remaining 5 could not participate for using phones registered under different names. Eventually 59 students took part in this study of whom 36 (61%) were females and 23 (39%) males. Mean age (standard deviation) was 13.0 (0.5) years.

### Self reported and logged use of mobile phones

**Figure 1:**
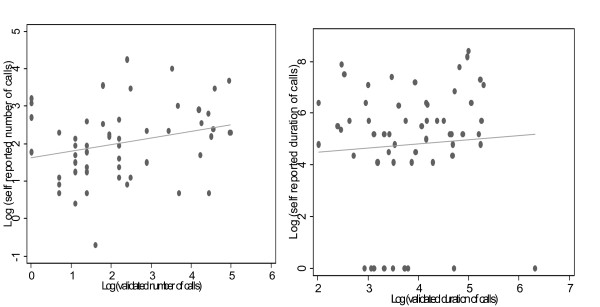
**Agreement between self reported and validated call indices per week: (a) number of calls (b) duration of calls**.

The agreement between self-reported mobile telephone use and actual phone use is presented in figure 1 comparing the level of agreement between self reported phone use in terms of number and duration of calls per week. Overall there appeared to be a modest correlation between self reported and actual phone use as logged by SMP (ρ = 0.3, P = 0.04) based on recall of number of calls. Agreement between SMP recorded and self reported duration of calls was poorer (ρ = 0.1, P = 0.37). Participants whose parents belonged to the 4^th ^socio-economic quintile recalled phone use better (ρ = 0.6, P = 0.01). These results and other possible predictors of agreement are presented in table 2.

**Table 2:** Possible factors influencing recall of mobile telephone use

Predictors		Call number		Call duration	
	**N**	ρ*	**P value‡**	ρ*	**P value ‡**

					

**Overall**	59	0.3	0.04	0.1	0.37

**Gender**					

Females	36	0.3	0.08	0.1	0.64

Males	23	0.2	0.50	0.2	0.37

Age					

<13	30	0.2	0.30	0.02	0.90

≥ 13	29	0.3	0.07	0.1	0.56

**Parental risk perception**					

No risk	4	0.3	0.68	-0.8	0.20

Low risk	12	0.3	0.40	0.6	0.02

Moderate/high risk	7	0.3	0.48	-0.1	0.80

Don't know	36	0.3	0.12	0.1	0.50

**Language other than English spoken at home**					

No	44	0.2	0.10	0.3	0.06

Yes	15	0.3	0.24	-0.4	0.13

**School system**					

Catholic	10	0.5	0.20	0.1	0.74

Independent	13	0.3	0.37	0.1	0.64

Government	36	0.2	0.29	0.1	0.60

**Socioeconomic status**					

Most disadvantaged	6	0.4	0.47	-0.4	0.47

2^nd ^quintile	2	1		0.7	0.01

3^rd ^quintile	12	0.2	0.80	0.2	0.48

4^th ^quintile	16	0.6	0.01	0.3	0.70

Most advantaged	23	0.05	0.80	-0.03	0.90

*Spearman's correlation coefficient

‡ P value – self reports and logged results are independent

**Figure 2:**
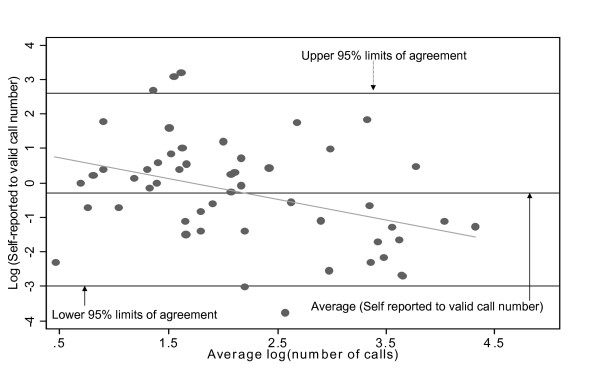
**Bland & Altman plot of ratio of self-reported to valid number of calls per week versus average of self-reported and valid number of calls per week**.

The recall of phone use was investigated in terms of sensitivity, specificity, positive predictive and negative predictive value using SMP records as the gold standard. The overall sensitivity of self reports was 57% and specificity was 66%. These results and others including key determinants are presented in table 3. There was little effect of age or ethnicity upon test properties. The level of agreement between recalled and validated number of calls was further assessed as a ratio of the two measures using the Bland & Altman plot. The 95% prediction interval (-3 to 2.6) is presented in figure 2. A ratio of 1 (or log ratio = 0) would indicate perfect agreement between self report and validation records. A summary of exposure metrics is presented in table 4.

**Table 3:** Agreement between SMP (gold standard) and self reported results

	N	Sensitivity (%)	Specificity (%)	PPV* (%)	NPV* (%)
**Overall**	59	57	66	63	41

**Gender**					

Females	36	58	65	65	42

Males	23	55	67	60	38

**Age**					

< 13	30	53	67	62	41

≥ 13	29	60	64	64	40

**Language other than English spoken at home**					

No	44	55	68	63	40

Yes	15	63	57	65	43

*PPV positive predictive value of self reports as percentage

*NPV negative predictive value of self reports as percentage

**Table 4:** Summary of exposure

	N**	SMP measured	Self reported
		Mean (SD*)***	Mean(SD)

**Number of calls**	59	2.4(1.3)	1.9(1.1)

**Duration of calls**	59	3.9(0.9)	4.9(2.2)

		Median(95% CI ‡)***	

**Number of calls**	59	2.3(1.6, 2.5)	2.1(1.7, 2.4)

**Duration of calls**	59	3.8(3.5, 4.2)	5.2(4.8, 5.7)

** Number of participants in the study

* Standard deviation

‡ 95% confidence interval

***Log transformed data

## Discussion

This validation study is unique in several ways: These are the first results validating adolescent recall of mobile telephone use. It is also the first report on the use of SMPs in this age group. Importantly this is the first independent report investigating accuracy of mobile telephone self reports in a sample representative of the main study. This study supports previous findings in adults that mobile phone recall correlates better with number of calls than duration of call[30,32]. We found a modest correlation between self reported and validated number of calls, but a weaker correlation between self reported and validated duration of calls. These findings are not entirely surprising. Mobile telephone users in this cohort have been previously reported to react faster at cognitive function tests but made more mistakes[36] compared to non mobile telephone users.

In this validation study, we have calculated sensitivity, specificity as well as positive and negative predictive values using SMP records as the gold standard in adolescents. Reducing the data to dichotomous simplifies the interpretation of results and makes for an easier understanding of the limitations of self-reports. This approach is appropriate as increasing numbers of non epidemiologists are reading the RF epidemiological literature.

A recent Danish study[29] pooled case-control and retrospective cohort study data and compared agreement between subscriber records and self reports with the latter as gold standard. A regular user in that study was defined as the use of mobile telephones at least once a week during a period of half a year or more during 1982–1995. Although based on such a low cut-point, only 19% of participants were regular mobile telephone users. In this validation study, we set the cut point outside the validation data set. We defined as high exposed all participants equal to or above the median exposure in the main MoRPhEUS study. Low exposure was exposures below this median. This approach is similar to a recent German study on exposure assessment and lung function in farmers[41]. In that study the authors compared the results of lung function for farmers with occupational exposures less than the median with those with occupational exposures greater than or equal to the median.

A recent international case control study investigating possible associations between mobile telephone use and head and neck tumours in adults[42] conducted some national validation studies[30-32,43] and a pooled analysis of results from 12 participating centres from 11 countries[25]. Almost all centres in this international validation study recruited convenience samples comprising colleagues, friends and acquaintances of investigators, except for the Australian and Northern UK centres. The Australian centre recruited from controls, who also expressed enthusiasm about participation in further research, but might not be representative of the original case-control study. Also the northern UK group utilised operator data from volunteers recruited through advertising in local newspapers, local council and to university staff. Furthermore, the New Zealand component recruited 35 to use the SMP for validation, however 43% of these were subsequently excluded from analysis for various reasons including SMP errors that prevented matching of participants to data. In our study we instituted the quality control regime listed in table 1 and did not suffer data or phone losses, albeit in a much younger population.

It is often convenient for researchers to recruit from colleagues and acquaintances in most health studies exploring adverse effects of common exposures to agents including mobile telephone use. A major drawback in such an approach has been the non representativeness of handpicked participants to the cohort under investigation. We have demonstrated that although seldom applied in wider epidemiological studies, it is practical to randomly recruit validation participants from the main study.

Our study has some limitations. Although we were conscious of and took practical steps to avoid selection bias, we cannot with certainty rule out the possibility of such influence on the outcome of this study. Contributing to selection bias was the nature of the SMP which effectively excluded 3G mobile phone owners from participation. Also as no incentives were offered to participants, it is possible that adolescents who volunteered to swap their better looking phones for the SMP for the long period of one week may be unique in some ways. We were not able to further investigate possible unique characteristics of participants and 3G users as compared to non participants.

A further limitation would involve the integrity of adolescent self reports. A recent study[28] found that the truthfulness and accuracy of adolescent self reports may be genuinely compromised by the difficulty of such recall. Furthermore this age group sometimes manipulates self reports depending on the perceived social acceptability or otherwise of the health behaviour under investigation. An example of adolescent self reports was recently demonstrated in a study which found body mass index (BMI) calculated based on adolescent self reported height and weight underestimated actual BMI calculated based on investigator measured height and weight [44].

Establishing a widely accepted validation instrument is technically difficult, but more so in an emerging field such as mobile telephone epidemiology. In this study, we have not only demonstrated the use of SMPs to validate adolescent self reports, but also described for the first time possible predictors of agreement between self reports and valid phone use in this young population. Our finding of a strong positive and significant correlation among students whose parents belonged to one of the higher socioeconomic strata was unexpected and this might be due to chance. Furthermore the wide confidence limit in the Bland and Altman plot (figure 2) could suggest a discrepancy between the two measures. Future studies involving this age group should also consider a brief interview with potential participants who chose not to participate. Such information is needed to further understand the determinants of adolescent participation in health studies generally. Also to improve the generalizability of results and increase sample sizes, we suggest recruitment of participants with a wider range of ages from both urban and rural schools.

## Conclusion

The overall very modest agreement between recalled and validated phone use in this study would argue for caution in the interpretation of results of epidemiological studies investigating health effects of mobile telephone use generally, but particularly in adolescents.

## Competing interests

Imo Inyang and Geza Benke declare no competing interests. Michael Abramson holds shares in Telstra and SingTel which operate mobile telephone networks in Australia. Joseph Morrissey was an employee of Motorola Inc at the time of the study. Ray McKenzie is an employee of, and holds shares in Telstra.

## Authors' contributions

Funding for MoRPhEUS study was obtained by GB and MA. Data collection was undertaken by RM and II. JM provided the SMPs as well as technical assistance with this validation instrument. Data analysis was performed by II and GB under the supervision of MA. All authors have participated in writing the manuscript and approving the final version for publication.

## Appendix 1: Inclusion criteria

(i) All participants were year 7-early secondary school students

(ii) Participants of Mobile Radiofrequency Phone Exposed Users Study (MoRPhEUS)

(iii) Only registered owners of mobile phones were included. Users but non phone owners including those who own phones but registered under different names were excluded

(iv) Only non third generation (3G) phone owners were included. SMPs were not 3G compatible

(v) Only participants with valid parental and self consent were included

## Pre-publication history

The pre-publication history for this paper can be accessed here:

http://www.biomedcentral.com/1471-2288/9/36/prepub
